# Exposure to indoor air pollution across socio-economic groups in high-income countries: A scoping review of the literature and a modelling methodology

**DOI:** 10.1016/j.envint.2020.105748

**Published:** 2020-10

**Authors:** Lauren Ferguson, Jonathon Taylor, Michael Davies, Clive Shrubsole, Phil Symonds, Sani Dimitroulopoulou

**Affiliations:** a UCL Energy Institute, Bartlett School of Environment, Energy and Resources, University College London, UK; b Institute for Environmental Design and Engineering, Bartlett School of Environment, Energy and Resources, University College London, UK; c Air Quality & Public Health Group, Environmental Hazards and Emergencies Department, Centre for Radiation, Chemical and Environmental Hazards, Public Health England, Harwell Science and Innovation Campus, Chilton, UK

**Keywords:** Indoor air quality, Household air pollution, Socio-economic status, Environmental justice, Indoor environment modelling, Preferred reporting items for systematic reviews and meta-analyses (PRISMA)

## Abstract

Disparities in outdoor air pollution exposure between individuals of differing socio-economic status is a growing area of research, widely explored in the environmental health literature. However, in developed countries, around 80% of time is spent indoors, meaning *indoor* air pollution may be a better proxy for personal exposure. Building characteristics - such as build quality, volume and ventilation - and occupant behaviour, mean indoor air pollution may also vary across socio-economic groups, leading to health inequalities. Much of the existing literature has focused on inequalities in exposure to outdoor air pollution, and there is thus a lack of an evidence base reviewing data for *indoor* environments. In this study, a scoping review of the literature on indoor air pollution exposures across different socio-economic groups is performed, examining evidence from both monitoring and modelling studies in the developed world. The literature was reviewed, identifying different indoor pollutants, definitions for socio-economic status and pre- and post- housing interventions. Based on the review, the study proposes a modelling methodology for evaluating the effects of environmental policies on different socio-economic populations. Using a sample size calculation, obstacles in obtaining sufficiently large samples of monitored data are demonstrated. A modelling framework for the rapid quantification of daily home exposure is then outlined as a proof of concept. While significant additional research is required to examine inequalities in indoor exposures, modelling approaches may provide opportunities to quantify exposure disparities due to housing and behaviours across populations of different socio-economic status.

## Highlights

• Households of low socio-economic status generally experience poorer indoor air quality.
• Higher radon concentrations were found in homes with a greater material wealth.
• Building modifications may worsen indoor air quality without appropriate ventilation.
• Models can analyse the effect of building modifications on indoor air quality.

## 1 Introduction

The presence of harmful substances such as gases, particulates or biological molecules in the Earth’s atmosphere is known as *air pollution* (Loomis et al., 2013). Human exposure to air pollution has serious implications for health: Short term exposure may exacerbate asthma and be responsible for hospital admissions (Zheng et al., 2015), whilst long term exposure to ambient air pollution is repeatedly associated with a higher incidence of cardiovascular and respiratory diseases (Pope et al., 2011, Atkinson et al., 2016, COMEAP, 2018a), birth defects (Padula et al., 2013) and neuro-degenerative disorders (Moulton and Yang, 2012). The Committee on the Medical Effects of Air Pollution (COMEAP) have estimated that ambient air pollution is responsible for between 28,000 and 36,000 deaths each year in the UK (COMEAP, 2018b). While there is a significant body of international research highlighting evidence of the association of areas of low socio-economic status (SES) with high levels of outdoor air pollution (Clark et al., 2014, Milojevic et al., 2017, Pinault et al., 2016, Fairburn et al., 2019), there is little on equivalent exposures to indoor air pollution, despite populations in developed countries spending the majority of their time indoors. The indoor environment is overlooked in the environmental health discourse, despite the considerable health risks that can arise (Bernstein et al., 2008). Thus, understanding variations in population exposure to air pollution across socio-economic groups, in both indoor and outdoor environments, is critical in reducing existing and future health inequalities.

Environmental health equity is the equal distribution of environmental risks across populations, whereby disadvantaged sub-groups are not disproportionately exposed to environmental externalities or have reduced access to natural amenities (WHO, 2019). A recent report published by the European Environment Agency (EEA) highlighted how environmental hazards, such as air pollution, noise and extreme events are unevenly distributed across populations within Europe, with increased risk associated with areas of lower SES (EEA, 2019). Environmental hazards typically have the greatest impact upon vulnerable populations within society due to their limited resources or ability to adapt to challenging conditions (Murage et al., 2020). People of lower SES are at increased risk of exposure to outdoor air pollution, noise and overheating, whilst children, older populations and those with chronic illness are more likely to experience adverse health effects from such exposures (EEA, 2019). These two mechanisms lead to differences in risk because 1) disadvantaged individuals within a wider population may be exposed to higher levels of environmental hazards, and 2) disadvantaged individuals have a greater vulnerability to adverse health effects from exposure due to underlying health conditions (Milojevic et al., 2017).

### 1.1 Outdoor exposures

In the developed world, the association of high outdoor air pollution concentrations with socio-economically vulnerable communities has been demonstrated in a selection of studies. Recently, Samoli et al. (2019) found that unemployment rate and population density were significant predictors of NO_2_ exposure in nine metropolitan areas across Europe. Research across Canada echoed this relationship, finding that children from homes in the lowest income quintiles were exposed to higher average NO_2_ levels (Pinault et al., 2016). In the UK, areas of high deprivation have been associated with elevated levels of PM_10_ across London (Tonne et al., 2008), Birmingham and Belfast (Pye et al., 2001). A systematic review of 31 papers across Europe found that elevated levels of both particulate matter (PM_2.5_, PM_10_) and nitrogen oxides (NO_x_) fell disproportionately on those of lower SES (Fairburn et al., 2019). Wide recognition of the health impacts associated with exposure to outdoor air pollution has led to improvements across much of the developed world (Clark et al., 2017), but environmental health inequalities can persist as mitigation strategies often target whole populations or specific areas which breach guidelines without directly aiming policies at the communities subject to disproportionate levels (EEA, 2019). A recent high-profile report by the World Health Organisation (WHO) on environmental health inequalities reported that health inequalities arising from exposure to outdoor air pollution were increasing in some areas of Europe despite air quality improvements (WHO, 2019).

### 1.2 Indoor exposures: The role of buildings and occupant behaviour

Ambient outdoor concentrations only offer a broad indication of personal exposure, which is likely to be determined by an individual’s time-activity profiles including time spent in indoor environments (Ashmore & Dimitroulopoulou, 2009). Reliance on outdoor concentrations may lead to exposure misclassification (Zipprich et al., 2002, Delgado-Saborit, 2012). Despite around 80% of modern life being spent indoors (Poljansek et al., 2017), the indoor environment is rarely included in the environmental equity dialogue (Adamkiewicz et al., 2011).

Buildings may significantly modify exposure to air pollutants originating from both indoor and outdoor sources (Taylor et al., 2014). Indoor air pollution from outdoor sources may occur due to infiltration of pollution from anthropogenic activities, such as vehicular traffic, a common source of particulate matter (PM) and nitrogen dioxide (NO_2_) (COMEAP, 2018), or natural sources, such as radon from radioactive decay in the ground (Turk et al., 1990). The airtightness of the dwelling, the number of external façades and their exposure to wind, and window-opening behaviour by the occupants will impact the amount of pollution that passively enters (Hänninen et al., 2004). In buildings with mechanical ventilation, active infiltration may also occur via these systems, with the infiltration rate dependent on the ventilation rate and presence of filtration systems. Indoor activities that generate air pollution include NO_2_ and PM_2.5_ from cooking and PM_2.5_ from solid fuel heating or smoking (Klepeis and Nazaroff, 2006, Géhin et al., 2008, O'Leary et al., 2019). Cleaning activities are a common source of indoor VOC emissions from aerosols and solvents (Dumanoglu et al., 2014, Dimitroulopoulou et al., 2015). Activities may vary in terms of presence, source intensity, frequency, and duration across SES.

Once inside, indoor pollutants may undergo transformation by chemical reactions on indoor surfaces. Reaction of NO_2_ with indoor surface materials can lead to the formation of nitrous acid (HONO), a pollutant with known health impacts (Gligorovski, 2016, Ma et al., 2017). The ventilation rate of the dwelling (either passive or active), internal deposition, and air filtration systems act as air pollution *sinks* and remove indoor pollutants. Housing characteristics such as airtightness, vents, purpose-provided ventilation systems, internal and external geometry, and occupant behaviours such as the ability to open windows for ventilation, impact the level of outdoor infiltration and may also vary between socio-economic groups (Taylor et al., 2014).

The aforementioned report by the EEA identified high quality housing as an effective response to improving unequal environmental exposures across Europe (EEA, 2019), but assembling of the scientific literature concerning indoor exposure disparities is needed to highlight populations who bear a disproportionate amount of the environmental burden. One of the mechanisms through which social inequality translates to health inequalities is via the quality of housing conditions, in which home environmental exposures, such as indoor air pollution, play a role (Braubach et al., 2009).

Policy-mediated changes to the built environment can lead to unintended consequences on occupant health (Shrubsole et al., 2014) via the dichotomy between increased energy efficiency and indoor air quality (Shrubsole et al., 2016, Broderick et al., 2017). Given their limited resources to adapt to changing conditions, those of low SES may be disproportionately affected by the unanticipated effects of housing improvement policies which are implemented without consideration of the wider socio-economic processes governing the space (Shrubsole et al., 2016). Identifying those at risk of high indoor concentrations can lead to better-targeted interventions, such as housing improvements, in the indoor environment.

### 1.3 Motivation for the work and objectives

While there have been a number of reviews on outdoor air pollution exposures across different SES groups (Deguen and Zmirou-Navier, 2010, Hajat et al., 2015, Fairburn et al., 2019), there is little research summarising the existing literature on how indoor exposure to air pollution varies across socio-economic groups in developed countries. To address this gap, a scoping review was carried in compliance with the PRISMA methodology for Scoping Reviews (Tricco et al., 2018). Our aims are listed below:

1. Investigate whether disparities in exposure to indoor air pollution exist for different socio-economic groups, evaluating the availability of the literature exploring indoor exposure disparities relative to outdoor air pollution;
2. Use (1) to illustrate the difficulty in acquiring monitored data enabling conclusions to be drawn about IAQ disparities and its drivers;
3. Describe a modelling approach that, while acknowledging the limitations and uncertainties, may help understand the disparities and their causes, forming the basis for health impact calculations.

As this is a relatively unexplored area of research, a scoping review was deemed the most appropriate review protocol to gauge the available literature and examine the variety of research methods. We sought to identify the socio-economic indicators used, as this is likely to differ from individual - or area-level measures of SES used in the outdoor literature – and investigate which indoor pollutants are commonly used as indicators of poor air quality in the *indoor* environment. Whilst acknowledging that indoor air pollution is a critical concern in developing nations, especially in homes with solid fuel use (e.g. Bruce et al., 2000, Smith and Mehta, 2003, Goldemberg et al., 2018), the current review focuses on developed countries only. The large heterogeneity in national circumstances that exists between the developed and developing world would make any comparison between the two unproductive without consideration of the wider state infrastructure, which was not the focus of this work.

The purpose of the research is to incorporate the indoor environment into the environmental equity literature and inform future housing policies regarding the role of socially-patterned housing characteristics and occupant behaviours on IAQ in the developed world. Different methods of data collection used to capture indoor exposures identified from the review are discussed, considering the drawbacks and benefits of each approach to guide future research. Finally, obstacles in attaining sufficiently large samples for monitoring studies are discussed and a modelling framework is outlined, which allows for the estimation of daily exposure to air pollution in indoor environments across a population. IAQ modelling techniques can help reconcile the large evidence gap which exists between indoor and outdoor air pollution studies, but have rarely been employed to examine exposure disparities between socio-economic groups. Quantifying diurnal variations in indoor exposures can help to evaluate the effect of potential interventions and identify the drivers of poor indoor air quality (IAQ) in low-SES homes, helping to target indoor air quality policies accordingly, improving population health and reducing inequalities in the developed world.

## 2 Methods

### 2.1 Scoping review

A comprehensive search of the literature was carried out in PubMed, SCOPUS and Web of Science. The inclusion criterion was as follows*: All relevant publications written in English from the year 2000 up until April 2019, carried out in the developed world*.^1^

The search terms are outlined in Table 1. In order to identify the types of available evidence in this field, no restrictions were placed on the species of air pollutant for which indoor exposure was estimated. Thus, no pollutants were specifically excluded, in order to assimilate all the available evidence and identify potential trends in the literature.

**Table 1:** Search Parameters**.**

**Key terms**	- *indoor air pollution* - *household air pollution* - *predictors of indoor air quality* - *socio-economic status* - *deprivation* - *lifestyle factors*

**Inclusion criteria**	- written in English
	- published after 2000
	- research conducted in the developed world
	- has been peer reviewed
	- concerns socio-economic factors which influence levels of indoor air pollution exposure
**Post-hoc exclusion criteria**	- studies which quantify exposure in low socio-economic households without a control population as a reference
	- studies which use race or ethnicity as the dependent variable instead of a socio-economic indicator
	- studies where socio-economic status is the effect modifier on a health outcome without quantifying the exposure disparity
**Search string in SCOPUS**	( TITLE-ABS-KEY ( household ) OR TITLE-ABS-KEY ( residential ) OR TITLE-ABS-KEY ( indoor ) AND TITLE-ABS-KEY ( air AND pollution ) OR TITLE-ABS-KEY ( exposure ) AND TITLE-ABS-KEY ( income ) OR TITLE-ABS-KEY ( depriv* ) OR TITLE-ABS-KEY ( socio* ) OR TITLE-ABS-KEY ( inequalit* ) OR TITLE-ABS-KEY ( unequal ) )

Table 1 outlining the main search parameters and an example search string. Note that the advanced search feature was used in all databases.

Sources of evidence were accepted if they estimated exposure to a given pollutant or surrogate of poor air quality in two populations of different socio-economic circumstances. Proxies of SES were accepted, if they commented on the social (i.e. education) or economic (i.e. income) standing of a household. Studies which looked at indoor exposures according to race (Adgate et al., 2004) or ethnicity (Ferrero et al., 2017) alone were excluded. For methods of exposure estimation, both monitored and modelled indoor concentrations were accepted, along with participant-reported exposure and biomarkers of exposure - the principal criterion being that the exposure was taking place in the home, and not school (Batisse et al., 2017) or outdoors (Padilla et al., 2014).

Details regarding the study location, method of air quality assessment, sample size, the socio-economic metric and the data from which this was acquired, pollutant, overall study findings and the level of significance were recorded for each piece of evidence. This was to identify potential salient factors relating to the variables recorded. This data was acquired solely through the publication in print and any further appendices provided by the authors. The PRISMA methodology for Scoping Reviews checklist is included in the appendix.

## 3 Results

**Fig. 1:**
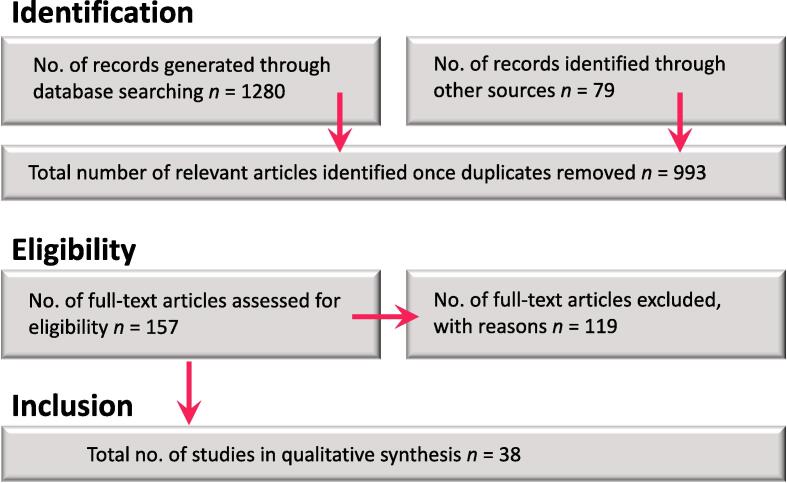
Schematic representation of the process used to identify literature.

A total of 38 publications were identified from the search and the process used to identify literature is outlined in Fig. 1.

The search yielded a large number of results (1280). Once duplicates were removed, the eligibility of articles was deduced from their title and abstract. An additional 79 papers were identified from citations within relevant studies, before the full-screening of 157 articles, excluding a total of 119, with the most common reasons for exclusion outlined in Table 1. No existing review was identified from the literature which specifically addressed the distribution of indoor exposures across socio-economic groups. However, a publication by Patino & Siegel (2018) reviewed the indoor environment quality in social housing. Whilst this work did not make direct comparisons between differentially deprived populations and exposure, it included measurements taken in a general setting to contextualise the findings. Though relevant to the work carried out in this review, there is no accepted definition of social housing and the term varies significantly across different countries, thus, these citations were not considered here.

A substantial part of the literature described the relationship between exposure to indoor environmental tobacco smoke and SES. Two reviews were identified in this area of research (Thomson et al., 2005, Orton et al., 2014), both of which cited SES as a determining factor in self-reported environmental tobacco smoke exposure in the home. Environmental tobacco smoke (ETS) is a primary source of PM_2.5_, NO_2_ and a number of volatile organic compounds (VOCs) (Arku et al., 2015, EPA, 2016), therefore research quantifying this association was deemed to meet the criteria of the scoping review but this literature has been presented separately due to the overwhelming number of publications when compared with the literature for individual air pollutants, (26 vs. 12). The majority of the literature reviewed in Thomson et al. (2005) was published before 2000, and therefore did not meet the criteria of our review. However, 21 of the 26 publications reviewed in the environmental tobacco smoke literature here have been previously reviewed by Orton et al. (2014). This review aims to build on the existing review (Orton et al., 2014) by framing the available evidence in an indoor air quality context, as opposed to determinants of childhood exposure to second-hand smoke, as per Orton et al. (2014).

### 3.1 Indoor air pollution across Socio-economic groups

Publications which explicitly characterised exposure to different indoor pollutants across socio-economic groups are outlined in Table 2, Table 3, Table 4, Table 5, and the influence of the various parameters employed to estimate the disparities are discussed in further detail under Section 3.2. In publications which assessed particulate matter, nitrogen dioxide and VOCs, all but one found statistically higher concentrations of indoor air pollution levels in households of lower SES. Conversely, studies monitoring radon showed households with *higher* SES were exposed to elevated levels (Casey et al., 2015, Kendall et al., 2016).

**Table 2:** Comparison of literature on exposure to indoor particulate matter (PM).

Study	Location	Air Quality Assessment	Sample Size	Socio-economic Data	Socio-economic Measure	Pollutant	Results	Significance
Baxter et al. (2007)	Boston, US	Home measurements.	43	Household questionnaire.	Household occupant density.	PM_2.5_	High household occupant density was associated with a 4.11 μg/m^3^ increase in indoor PM_2.5_ after adjusting for outdoor levels.	p=<0.2
Byun et al. (2010)	Korea: - Gyeonggi - Seoul (2 sites).	Home measurements.	50	Household questionnaire.	Educational attainment; Household monthly expenses.	PM_10_	PM_10_ decreased by 18.84 μg/m^3^ as parental education increased from high school or lower to college or higher. PM_10_ decreased by 6.61 μg/m^3^ as average monthly expenses increased.	Educational attainment: - p = **0.01** Monthly expenses: - NS^a^
Brown et al. (2015).	France, nationwide	Home measurements.	567	Household questionnaire.	Household income; Occupational status; Household occupant density.	PM_2.5_	PM_2.5_ concentrations between employed (38.8 μg/m^3^) and unemployed (62.1 μg/m^3^), those with equivalised income <€999 (46.1 μg/m^3^), >€2099 (37.1 μg/m^3^) and low occupant density (26.8 μg/m^3^) and high occupant density (40.0 μg/m^3^) was significantly different.	PM_2.5_ - Occupation: p=<**0.001** - Income: p=<**0.05** - Occupant density: p=<**0.05**
Shrubsole et al. (2016)	England & Wales, nationwide	Building simulation.	~16,000	English Housing Survey.	Household income.	PM_2.5_	All tenures (owner occupied, local authority, and private rented homes) experienced lower indoor PM_2.5_ concentrations than households below the low-income threshold (LIT).	p = **0.05**
Rosofsky et al. (2018)	Massachusetts, US	Linked 3 publicly available datasets: housing, demographic + meteorological to parametrise an air exchange rate (AER) equation.	177	Census data.	Educational attainment†; Household income†.	PM_2.5_	Household income: Building groups which contained building envelopes with the lowest ambient PM_2.5_ & lowest AER (=lowest indoor exposures) were comprised of just 7% of households where median annual income <$20,000, compared with 23% in homes with high indoor exposures. Education: Low exposure areas comprised of 5% of households where the head of house had less than a high school attainment, compared with 21% in areas of high indoor exposures.	Percentile analysis.
Stamatel-opoulou et al. (2019)	Athens, Greece	Home measurements.	13	Household questionnaire.	Maternal occupational status.	- PM_2.5_ - PM_10_	In houses with working mothers, mean concentrations of PM_10_ and PM_2.5_ during weekdays were 21.7 and 10.1 μg/m^3^, respectively. In houses with unemployed mothers, the equivalent concentrations were 22.1 and 10.4 μg/m^3^, for PM_10_ and PM_2.5_ respectively.	PM_10_ - NS PM_2.5_ - NS

*values shown in bold were significant at the 95% confidence level.

a Not significant.

† Both at the block group (BG) level.

**Table 3:** Comparison of literature on exposure to indoor NO_2_.

Study	Location	Air Quality Assessment	Sample Size	Socio-economic Data	Socio-economic Measure	Pollutant	Results	Significance
Zota et al. (2005)	Boston, US	Home measurements.	77	Household questionnaire.	Household occupant density.	NO_2_	Occupant density was a significant predictor of indoor NO_2_ concentrations, with a univariate coefficient of 3.2.	p = 0.01*
Esplugues et al. (2010)	Valencia, Spain	Home measurements.	352	Study questionnaire, (INMA study)^a^.	Mother’s educational attainment.	NO_2_	For mothers with a primary education or lower, indoor NO_2_ levels were 0.07 μg/m^3^ higher than in the homes of mothers with a university education.	p = **0.04**

*values shown in bold were significant at the 95% confidence level.

a Ribas-Fitó et al. (2006).

**Table 4:** Comparison of literature on exposure to radon.

Study	Location	Air Quality Assessment	Sample Size	Socio-economic Data	Socio-economic Measure	Pollutant	Results	Significance
Casey et al. (2015)	Pennsylvania, US	Building measurements in the basement and 1st floor of multi-unit housing.	762, 725	Census data.	Building deprivation index.	Radon	Geometric mean radon concentration was 118.4 Bq/m^3^ in the basements of buildings in the lowest deprivation category and 103.6 Bq/m3 in the highest area deprivation category (buildings in areas with the highest deprivation score had the *lowest* indoor radon concentrations).	p = 0.05*
Kendal et al. (2016)	Great Britain, nationwide	Home measurements.	3189	Interview data from UK Child Cancer Study (UKCCS)^a^.	Social class, derived from parental occupation.	Radon	Geometric mean indoor radon concentrations decreased from 29.4 Bq/m^−3^ to 18.4 Bq/m^−3^ as social class of parent decreased.	None reported - recorded absolute measurements only.

*values shown in bold were significant at the 95% confidence level.

a UKCCS (2000).

**Table 5:** Comparison of literature on exposure to indoor VOCs.

Study	Location	Air Quality Assessment	Sample Size	Socio-economic Data	Socio-economic Measure	Pollutant	Results	Significance
Son et al. (2003)	Korea: - Asan - Seoul	Home measurements.	60	Household questionnaire.	Household income.	VOCs: - Benzene - Toluene - *o*-Xylene	Benzene Low-income = 78.90 μg/m^3^Other = 16.43 μg/m^3^ Toluene Low-Income = 211.1 μg/m^3^Other − 85.97 μg/m^3^*— o-*XyleneLow-income = 71.71 μg/m^3^Other = 39.21 μg/m^3^	Benzene - p = 0.013* Toluene - p = **0.020** *o-*Xylene - p = **0.001**
Storm et al. (2013)	New York, US	Home measurements.	126	Household questionnaire.	Income (5 categories).	VOCs (perchloroet-hylene)	Mean indoor concentrations were six times higher in homes in the lowest income category (105.5 μg/m^3^) compared with the highest (17.8 μg/m^3^).	p = **0.05**
Brown et al. (2015).	France, nationwide	Home measurements.	567	Household questionnaire.	Household income; Profession; Household occupant density.	VOCs (BTEX, formalde-hyde)	BTEX increased by a factor of 1.17 between household occupant density categories (low, med, high) in regression model. For regression model, lower income and higher occupant density was a predictor of higher formaldehyde levels by a factor of 0.96 and 1.10, respectively. Having a skilled profession increased levels by a factor of 1.20.	BTEX: - Occupant density: p=<**0.05** Formaldehyde (all SES metrics): - p=<**0.05**
Stamatel-opoulou et al. (2019)	Athens, Greece	Home measurements.	13	Household questionnaire.	Maternal occupational status.	TVOCs^a^	In houses with working mothers, the mean concentration of TVOCs during weekdays was 129.0 μg/m^3^. In houses with unemployed mothers, the equivalent concentrations were 62.7 μg/m^3^ for TVOCs.	TVOCs:- NS^b^

*values shown in bold were significant at the 95% confidence level.

^a^VOCs = Total volatile organic compounds.

^d^Not significant.

As outlined in Table 2, Table 3, Table 4, Table 5, home measurements were the most common method used to capture indoor levels and sample sizes varied from 13 (Stamatelopoulou et al., 2019) to 3189 homes (Kendall et al., 2016)^2^. Casey et al. (2015) used building measurements collected from 762,725 individual buildings between the years 1989 and 2013, some of which were multi-unit dwellings, so the number of individual homes is likely to be much higher. For home sampling, a questionnaire was the primary instrument through which socio-economic information was attained. A distinction was made between a *study questionnaire* that was collected at the individual-level on individual variables alone (e.g. parental educational attainment) as part of a wider study, and a *household questionnaire* where a survey was distributed to a household collecting household-specific information such as household income or home occupant density. Questionnaires which collect sensitive information regarding personal characteristics, such as household income (Son et al., 2003, Brown et al., 2015, Shrubsole et al., 2016, Rosofsky et al., 2018), may be especially vulnerable to social desirability bias, encouraging answer falsification. In studies with relatively large sample sizes, such as 3189 homes (Kendall et al., 2016), inaccurate answers are assumed to have a negligible effect on the results, but caution must be applied in those with smaller sample sizes.

#### 3.1.1 Particulate matter

Exposure to both PM_10_ and fine particulate matter (PM_2.5_) were assessed between socio-economic groups across papers in the review. Results are outlined in Table 2. PM_10_ and PM_2.5_, are defined as the fraction of particles with an aerodynamic diameter smaller than 10 and 2.5 µm, respectively, which pass through a size-selective inlet with a 50% efficiency (ISO, 2009). These are the thoracic and respirable fractions capable of passing beyond the larynx and ciliated airways, respectively, during inhalation (CEN, 1993). Two of the papers outlined in Table 2 estimated indoor PM_2.5_ concentrations using modelling techniques (Shrubsole et al., 2016, Rosofsky et al., 2018). Due to the difficulties directly comparing monitored data with modelled estimates, these studies are discussed elsewhere in Section 3.2.2. on IAQ modelling.

For empirical data, significantly higher levels of particulate matter were recorded in homes with higher household occupant density (Baxter et al., 2007, Brown et al., 2015), lower household education (Byun et al., 2010), lower household income (Brown et al., 2015) and unemployment (Brown et al., 2015). Household occupant density is an expected predictor of elevated indoor levels of PM due to the resuspension of particles that occurs from occupant movement (Klepeis & Nazaroff, 2006). No relationship was found between PM and SES in Stamatelopoulou et al. (2019), but this likely reflects the SES metric used. Here, maternal occupational status was used as a proxy for household SES to assess the relationship between indoor PM_2.5_ and VOC exposures (Stamatelopoulou et al., 2019). The suitability of this indicator as a measure of household SES is discussed below in Section 3.2.3.

A common source of indoor PM is the incidence of indoor smoking (Arku et al., 2015). Smoking itself is a strongly socially-patterned behaviour (NHS, 2019), and it is possible that the underlying smoking rates between socio-economic groups cause disparate indoor levels. Further, there has been an uptake of indoor solid fuel use across Western Europe (Fuller, 2019) and open combustion in fire places is the predominant source of outdoor PM_2.5_ levels in the UK (DEFRA, 2019, McGrath et al., 2017). In addition, cooking activities, which can vary between ethnic minority groups and practices, especially in the absence of proper extractor fans, can contribute to high PM levels (Abdullahi et al., 2013, O'Leary et al., 2019). Poorer quality housing may struggle to disperse indoor concentrations of PM_2.5_ via inefficient ventilation.

##### 3.1.1.1 Nitrogen dioxide

High levels of NO_2_ can accumulate in cities due to emissions from vehicles and all three publications which looked at indoor NO_2_ recorded levels in urbanised areas of Boston (Zota et al., 2005, Baxter et al., 2007), a large metropolis in Massachusetts, US, and Valencia, the third largest city in Spain (Esplugues et al., 2010). No socio-economic metric was considered as a covariant for NO_2_ in Baxter et al. (2007) and this publication is therefore omitted from Table 3 below, but the study found that the outdoor NO_2_ concentrations were a significant predictor of indoor NO_2_ levels, suggesting infiltration of outdoor-sourced air pollution may be in-part responsible for indoor exposures. High outdoor pollutant concentrations are often a proxy for areas of low SES, as location near congested roads can cause land price to depreciate, attracting purchase by lower-income individuals and local councils for social housing (Deguen & Zmirou-Navier, 2010).

In studies which considered an SES metric as a predictor of indoor NO_2_ exposure, low educational attainment was associated with a 0.7 µg/m^3^ increase in indoor NO_2_ concentrations (Esplugues et al., 2010) and household occupant density increased NO_2_ levels three-fold (Zota et al., 2005). High density is associated with higher concentrations of air pollutants, both in terms of indoor occupancy (Zota et al., 2005, Baxter et al., 2007) and outdoor population density (Samoli et al., 2019). For air pollutants primarily produced by anthropogenic activities such as NO_2_, a greater number of occupants (or a larger population) is conducive to higher incidence of the human-generated events which are common sources of NO_2_, such as longer cooking times to accommodate for a larger household size (Singer et al., 2017) or higher traffic volumes in densely populated areas (Samoli et al., 2019).

##### 3.1.1.2 Radon

Two publications from the literature found indoor radon concentrations were higher in homes with greater material wealth (Casey et al., 2015, Kendall et al., 2016), as shown in Table 4. While the presence of radon in homes is principally explained by geological variables (Jacobs, 2011), dwelling characteristics also play a role. The primary mechanism of radon-entry in homes is via the pressure-driven diffusion from the soil in contact with the ground floor of buildings (Turk et al., 1990). The rate of infiltration from the soil is dependent on the surface area of the ground floor, cracks within that exchange surface and temperature differences between indoor and outdoor air (Turk et al., 1990). Those of low socio-economic status are more likely to live in smaller dwellings, such as flats with smaller floor areas (Taylor et al., 2014), where only ground-floor properties will be at high risk of radon infiltration from the ground. Higher internal temperatures may be indicative of more affluent homes (Palmer & Cooper, 2013) and exacerbate the rate at which gases from the soil beneath can diffuse into the home by increasing the pressure gradient (Turk et al., 1990). Further, energy-efficient features, such as double glazing, wall and loft insulation are associated with higher indoor radon levels (Symonds et al., 2019), as dwellings with a tight building envelope may struggle to disperse indoor concentrations causing levels to accumulate. As housing quality is often indicative of material circumstance, better dwelling airtightness may be resulting in households of higher socio-economic status exposed to elevated indoor radon concentrations, along with higher internal temperatures.

##### 3.1.1.3 Volatile organic compounds

Three out of the four studies on VOCs found evidence that those of lower SES are exposed to elevated levels of indoor VOCs and results are displayed in Table 5. However, Son et al. (2003) suggested socio-economic differences in indoor exposures to benzene, toluene and *o*-xylene may have been an artefact of geographical factors: The majority of low-income housing was located in Seoul, the most densely populated city in Korea, where ambient pollutant concentrations are higher than the national average (Park et al., 2013). As outdoor levels are highly correlated with indoor concentrations, the high proportion of average income housing in Asan, a city with a population 1/50th of Seoul, may have benefitted from the infiltration of cleaner outdoor air (Son et al., 2003). Benzene, toluene and *o*-xylene are often emitted by the same sources and, along with ethylbenzene, collectively referred to as *BTEX* compounds (D'souza et al., 2009). Emission by solvents and paint coatings are common indoor sources of BTEX compounds, while outdoor sources include vehicle exhausts (D'souza et al., 2009). Thus, the higher traffic volumes in the highly-urbanised area of Seoul may be contributing to the infiltration of poorer air quality experienced by those living in low income housing in the capital city (Son et al., 2003).

In other studies, formaldehyde levels were found to be higher in homes with highly skilled professionals (Brown et al., 2015). As concentrations increased for all other markers of low SES, the authors suggested the ubiquity of formaldehyde in the indoor environment makes it difficult to identify a trend but is likely related to the use of certain consumer products (Brown et al., 2015). In New York, higher perchloroethylene (PERC) concentrations in low income, multi-unit housing were attributed to the presence of a co-located dry cleaner on the ground floor of the buildings (Storm et al., 2013). The presence or use of local dry-cleaning services has been highlighted as a common source of elevated PERC exposures in a number of studies (D'souza et al., 2009, Wang et al., 2009). Further, Stamatelopoulou et al. (2019), monitored both particulate matter and VOCs across households, using maternal occupational status as the socio-economic metric, finding no significant difference in VOC levels in the homes of employed verses unemployed mothers. Further discussion regarding occupational measures of SES is provided below under Section 3.2.3.

##### 3.1.1.4 Environmental tobacco smoke

Environmental tobacco smoke is a primary source of PM_2.5_, NO_2_ and a number of VOCs (Arku et al., 2015, EPA, 2016). Significant associations were found between the presence of a smoker in the home and elevated levels of NO_2_ (Zota et al., 2005) and PM_2.5_ (Brown et al., 2015) in the literature outlined in Table 2, Table 3. Given the stark disparities which exist in the underlying smoking rates between socio-economic groups (ONS, 2017), it is plausible to expect the prevalence of indoor environmental tobacco smoke to mirror this socio-economic gradient: In the UK, 29% of unemployed adults smoke, compared with 15% of those employed (NHS, 2019). Exposure to ETS in the home is a leading environmental risk factor for asthma incidence in children (Noutsios and Floros, 2014, Simons et al., 2014), and is associated with lung cancer, cardiovascular disease and chronic obstructive pulmonary disease (COPD) and complications of the digestive system (PHE, 2018).

Much of the developed world has adopted national regulations banning smoking in public spaces with the principal aim of protecting the health of non-smokers (Schmidt, 2007). The home is yet to be incorporated into this legal framework and therefore remains a source of exposure for many adolescents and young children, despite shifting societal norms regarding smoking following the introduction of public smoking bans. Although the prevalence of smoke-free homes has increased along with changing attitudes towards smoking in the presence of children (Jarvis et al., 2009), exposure to second-hand smoke was associated with 22,600 new childhood asthma cases and 40 sudden infant deaths in the UK in 2010 (NICE, 2013). Young children born into households of low SES are disproportionately affected by passive smoking and bear an unequal amount of the health burden (NICE, 2013). Therefore, studies which assess home ETS-exposure are critical in evaluating the success of policies in achieving their primary aim of protecting the health of those most vulnerable. Thus, childhood home exposure to ETS was the primary dependent variable in all but one of the studies from the literature which quantified home exposure across socio-economic status, as outlined in Table 6.

**Table 6:** Comparison of literature on exposure to indoor ETS.

Study	Location	Air Quality Assessment	Sample Size	Socio-economic Data	Socio-economic Measure	Results	Significance
Mannino et al. (2001)	US, nationwide	Serum cotinine sample.	5653 children	Study questionnaire	Parental educational attainment; Family poverty index.	Low parental education led to 0.39 ng/ml increase in cotinine levels compared with higher educated parents. Those below the poverty index line had 0.18 ng/ml higher blood cotinine levels.	- Education: p < 0.05* - Poverty index: NS^a^
Berman et al. (2003)	Los Angeles, US	Parental-reported† home exposure, validated with home nicotine monitor.	242 children	Study questionnaire	Parental educational attainment.	Children with parents who had < high school education had elevated levels of indoor ETS exposure (130.5 h per week) vs. ≥ high school education (109.9 h).	**p < 0.05**
Jurado et al. (2004)	Granada, Spain	Parental-reported home exposure and urinary cotinine sample.	115 children	Study questionnaire	Parental educational attainment; Household occupant density	The higher the educational level of the father, the lower the cotinine levels in the child. Children living in a house with an occupant density of > 1 per bedroom had higher mean urinary cotinine than those with a household occupant density of < 1 per bedroom.	- Education: **p < 0.05** - Occupant density: **p = 0.036**
Scherer et al. (2004)	Ausburg, Germany	Parental-reported home exposure and urinary cotinine samples.	1220 children	Study questionnaire	Parental educational attainment.	Parental education was a significant predictor of home ETS exposure, with a partial *r*^2^ of 3.5. Children of parents with the lowest educational level had approximately 3x higher urinary cotinine levels than children of parents with the highest educational level.	- Reported exposure: **p < 0.01** - Cotinine samples:NS
Soliman et al. (2004)	US, nationwide	Parental-reported home exposure.	15,601 families	Study questionnaire	Parental educational attainment.	Children of mothers who were a high school drop out were almost four times more likely (OR^b^ = 1.18) to be exposed to ETS in the home than the children of mothers with a postgraduate education (OR = 0.28).	**p < 0.05**
Rise et al. (2005)	Norway, nationwide.	Parental-reported home exposure.	212 households	Study questionnaire	Parental educational attainment.	In 1995: parental education was a significant predictor of ETS exposure (β-coefficient = 0.17). 2001: parental education was again a significant predictor of home ETS exposure (β = 0.16).	- 1995: **p < 0.05** - 2001: **p < 0.05**
Gonzales et al. (2006)	New Mexico, US	Maternal-reported exposure.	269 mothers	Study questionnaire	Maternal educational attainment; Maternal occupational status.	No significant associations were found between educational attainment or occupational status and having a partial or no smoking ban at home.	- Education: p = 0.112 - Occupation: p = 0.572
Bolte et al. (2008)	Bavaria, Germany	Parental-reported home exposure.	12,422 children	Study questionnaire	Parental educational attainment; Parental occupational status. Household income.	Children of parents with a very high education had an adjusted odds ratio (OR) of 1.0 for ETS exposure, compared with very low education of 3.94. Children where both parents were unemployed/marginally employed were almost twice as likely to be exposed to ETS in the home (OR = 1.88) than those who had at least one employed parent (OR = 1.0). Homes with a household income of < 60% of the national median OR = 1.45, compared with homes with the national median household income OR = 1.0.	- Education: **p < 0.05** - Occupation: **p < 0.05** - Household income: **p < 0.05**
Hughes et al. (2008)	Seoul, Korea	Parental-reported home exposure.	207 parents	Verbal study questionnaire	Parental educational attainment; Parental occupational status.	35% of parents with < high school education reported home ETS exposure among children, compared with 26% of parents with > college education. 29% of white collar workers vs. 39% of blue collar workers reported childhood ETS exposure in the home.	- Education: p = 0.189 - Occupation: p = 0.581
Akhtar et al. (2009)	Scotland, nationwide	Self-reported home exposure and salivary cotinine sample.	2527 children	Study questionnaire	Family affluence scale^c^	Children from low affluence families (OR = 3.28) were>3 times more likely to have ‘‘no’’ restrictions over a ‘‘complete’’ smoking ban compared with high affluence families (OR = 1.0). Low FAS score was significantly associated with higher salivary cotinine concentration.	- Restriction: **p < 0.05** - Cotinine sample: **p < 0.001**
Mantziou et al. (2009)	Athens, Greece	Maternal-reported home exposure.	614 children	Study questionnaire	Parental educational attainment.	Lower educated fathers were *less* likely to expose their children to ETS in the house in comparison to their higher educated peers (OR = 0.57 vs 1.0).	p = 0.077
Akhtar et al. (2010)	Scotland, nationwide	Self-reported home exposure and salivary cotinine sample.	2389 children	Study questionnaire	Family affluence scale; Family SES^d^	Both low family affluence and SES was associated with a higher proportion of children reporting that one or more parents smoked in the home. Both SES and family affluence were significantly associated with salivary cotinine concentrations.	All regression models: **p < 0.001**
Alwan et al. (2010)	Leeds, UK	Parental-reported home exposure.	318 homes	Study questionnaire	Parental educational attainment; Parental occupational status.	Low parental education made it 2.2x more likely to smoke in the presence of a child. 53% of unemployed parents smoked in the presence of a child, compared with 37.4% of those employed.	- Education: p < **0.05** - Occupation: p = **0.017**
Singh et al. (2010)	US, nationwide.	Parental-reported home exposure.	90,853 children	Verbal study questionnaire	Parental educational attainment; Household poverty status^e^	Childhood exposure to ETS in the home was prevalent in 16.44% of households with the lowest paternal education (<12yrs), compared with 1.98% in households with the highest (>16yrs). Smoking occurred in 14.54% of homes with high poverty status, compared with 2.49% in homes with the least poverty.	- Education: **p < 0.01** - Poverty status: **p < 0.01**
Yi et al. (2011)	Korea, nationwide.	Parental-reported home exposure and urinary cotinine samples.	7059 children	Study questionnaire	Parental educational attainment; Household income; Area-level deprivation index	Those with low paternal education had higher odds of ETS exposure (OR = 1.81) than low maternal educational attainment (OR = 1.23), with the equivalent high parental educational group OR of 1.0. Children in homes with a low household income had higher odds of ETS exposure (OR = 1.28), than children in high income homes with (OR = 1.0). The most deprived areas had a higher odds ratio (OR = 1.34), compared with the least deprived areas (OR = 1.0)	- Education: **p < 0.05** - Income: **p < 0.05** - Area: **p < 0.05**
Pisinger et al. (2012)	The Capital Region, Denmark	Parental-reported home exposure.	21, 985 parents	Study questionnaire	Parental educational attainment.	OR of home exposure likelihood for children of parents with low education = 11.5, vs OR = 0 for high education: It was 11 times more likely for a child to be exposed to ETS at home if the parent had a very low education.	p < **0.001**
Ren et al. (2012)	Detroit, US	Maternal-reported home exposure.	399 children	Study questionnaire	Maternal educational attainment; Household income.	In a sample of mothers who didn’t smoke, 26% of those with a < high school education exposed their children to ETS, compared with 4% of those with > college education. No associations existed between childhood exposure to ETS and maternal household income.	- Education: p **< 0.01** - Household income: NS
Hawkins & Berkan (2013)	US, nationwide.	Maternal-reported exposure.	135,278 mothers	Study questionnaire	Maternal educational attainment.	13.4% of mothers with 16 + years of education smoked in the presence of infants for 1 + hours per day, compared with 28.1% of mothers with 0-11yrs education.	**p < 0.05**
Longman & Passey (2013)	Australia, nationwide	Parental-reported home exposure.	15,978 households.	Census data	Area-level deprivation index	Children living in areas in the lowest deprivation category were 4x more likely to be exposed to ETS in the home (OR = 1), than children in the highest category (OR = 0.25).	**p < 0.05**
Liao et al. (2014)	Taiwan, statewide	Parental-reported home exposure.	307 parents	Study questionnaire	Parental educational attainment; Parental occupational status; Household income.	55% of parents with > bachelor’s education and 75% of those with < junior high education smoked in the presence of children. 58% of parents with an annual income of >$600,000 smoked in the presence of children, compared with 72% of those where annual income = <$600,000. No associations were significant for occupational status.	- Education: **p < 0.05** - Household income = **p < 0.05** - Occupation: NS
Raisamo et al. (2014)	Finland, nationwide	Self-reported home exposure.	72,726 adolescents	Study questionnaire	Parental educational attainment.	Paternal educational attainment: in 12-14yo, those with a low paternal education were twice as likely to be exposed to ETS in the home. In 16-18yo, the equivalent group was 1.7x more likely to be exposed to home ETS. Maternal educational attainment: In 12-14yo low educational attainment was 2x as likely to be exposed to ETS in the home (OR = 2.3) than high education attainment (OR = 1.0) 16-18yo, low education OR = 1.7 vs. high OR = 1.0	Paternal education: - 12–14: NS - 16–18: **p = 0.002** Maternal education - 12–14: NS - 16–18: **p = 0.016**
Ulbricht et al. (2014)	Mecklenburg, Germany	Parental-reported home exposure.	3570 households	Study questionnaire	Parental educational attainment; Parental occupational status.	Indoor smoking was 5x more likely in households with parents with a low educational attainment (OR = 1), than those with high (OR = 0.19) Unemployed parents were 2.5x more likely to exposure their children to ETS in the home (OR = 2.72) than employed (OR = 1.0)	- Education: p **< 0.001** - Occupation: p **< 0.001**
Shiue (2015)	Scotland, nationwide	Parental-reported home exposure.	1019 children	Study questionnaire	Area-level deprivation index	Loose rules’ regarding indoor smoking were more prevalent in deprived areas vs. non-deprived areas.	**p < 0.001**
Kuntz & Lampert (2016)	Germany, nationwide	Parental-reported home exposure.	4455 parents	Study questionnaire	Household deprivation index	2006: 46.3% of children of low SES were exposed to ETS in the home, compared with 7.0% of high SES 2012: equivalent measures in 2012 were 19.4 vs. 1.7%	- 2006: **p < 0.001** - 2012; **p < 0.001**
Yao et al. (2016)	US, nationwide	Self-reported home exposure.	18,731 children; 44,049 adults	Study questionnaire.	Educational attainment; Poverty status.	2000: 35.1% of children with < high school educated parents were exposed to ETS in the home, vs. 9.0% in children who’s parents had a > college degree. 2010: equivalent figures were 9.4% and 8.2%, with no significant difference.	- 2000: **p < 0.001** - 2010: NS
Nguyen et al. (2018)	Japan, nationwide	Self-reported home exposure.	2,891 participants	Study questionnaire.	EA; OS; Household expenditure.	Women with < 9 years of education (OR = 2.37) had a higher risk of passive smoking at the home than women with > 13 years of education (OR = 1.0). Employed woman (OR = 1.44) had a higher chance of home passive smoking than unemployed women (OR = 1.0).	- EA: **p < 0.05** - OS: **p < 0.05** - Household expenditure: NS

†In studies which assessed childhood exposure to ETS, levels were determined by parental responses.

*values shown in bold were significant at the 95% confidence level.

a NS – not significant.

b OR – Odds Ratio

c FAS – Family Affluence Scale, derived from measures of car & computer ownership, household occupancy & family holidays.

d SES – Socio-economic status

e Household poverty status - measured as a ratio of family income to federal poverty level.

A number of publications conducted cross-sectional studies (Soliman et al., 2004, Rise and Lund, 2005, Akhtar et al., 2009, Akhtar et al., 2010, Pisinger et al., 2012, Liao et al., 2013, Raisamo et al., 2014, Kuntz and Lampert, 2016, Yao et al., 2016), all of which found reductions in the prevalence of home ETS exposure amongst children. Such studies emphasise the role of introducing national legislation in combating ETS exposure and protecting the health of those most vulnerable. Akhtar et al. (2010) found that reductions were most marked in populations of low SES, suggesting legislation can narrow the inequalities gap. However, results from Pisinger et al., 2012, Raisamo et al., 2014 were to the contrary, finding that reductions in the prevalence of exposure were the smallest for low socio-economic groups, suggesting that legislation may be better targeted at low-SES populations in order to reduce exposure and improve health inequalities. One study found the type of information parents receive is crucial in determining the success of a public smoking ban extending to the home environment and protecting childhood health: In 1995, Rise and Lund (2005) found that whether or not parents had been encouraged to establish household smoking rules significantly predicted childhood exposure along with the educational attainment of the parent. By 2001, whilst low-educational attainment was still significant, parental attitudes towards smoking were conducive of home exposure. The authors suggested this shift in strategies was indicative of the success of targeting intervention measures to individuals, such as information pertaining to parental attitudes, as opposed to structural interventions, such as imposing a home smoking ban without shifting of the underlying attitudes (Rise and Lund, 2005).

Despite 23 out of 26 of the papers reviewed in this section finding exposure disparities, there were some outliers to the rule: Berman et al., 2003, Mantziou et al., 2009 found that children with parents who had a higher educational attainment were exposed to elevated levels of indoor ETS, however for Mantziou et al. (2009) this was just short of the level of significance, at *p* = 0.077. The study by Berman et al. (2003) was carried out in Los Angeles, US and suggested that the findings were an artefact of cultural differences, as those with low educational attainment were more likely to be Latino-American and smoking prevalence was much lower in this subgroup of the population.

Age-related differences in exposure also emerged from the literature: Raisamo et al. (2014) found significant associations between socio-economic status and home childhood ETS exposure existed for only the older cohort of children (16–18 years old). Research by Singh et al. (2010) also found older children were more likely to be exposed to ETS in the home, potentially due to the increased awareness around the effects of ETS on infant respiratory systems.

### 3.2 The influence of data collection methods

#### 3.2.0.1 Air quality assessment

For exposure to indoor PM, NO_2_, radon and VOCs, home measurements were the most common method for estimating exposure, with only two studies modelling indoor concentrations. Whilst there are known issues with monitoring devices in homes, it was assumed the extent of uncertainty in the method of air quality assessment was confined to the reliability of the measuring equipment. Monitoring offers a way of safeguarding against problems associated with self-reported data, however it can be challenging due to the way occupants interact with monitoring equipment and the question of whether such data is sufficiently rich when used without any qualitative input (Foulds et al., 2013). Thus, whilst the use of sensor networks is commonly regarded as the most efficient way to capture indoor exposures, a robust methodology is yet to be defined.

Within the environmental tobacco literature, methods of data collection were the largest source of uncertainty, as 77% of studies used parental-reported home smoking prevalence to estimate the level of ETS exposure. Smoking in the presence of children has increasingly become a social taboo following the introduction of publicly-imposed smoking bans (Jarvis et al., 2009), thus parents may not have been forthright in revealing their smoking habits. Evidence of this in the literature was the presence of cotinine, the predominant nicotine metabolite, in the urine of 14% of children whose parents identified as a non-smoker (Jurado et al., 2004). However, Scherer et al. (2004) noted that, especially for children from low-SES backgrounds, the likelihood of coming into contact with smokers outside of their home is high. Other methods of exposure estimation included a home nicotine monitor (Berman et al., 2003) and childhood-reported home exposure (Akhtar et al., 2009, Akhtar et al., 2010, Yao et al., 2016). Difficulties can arise when using childhood responses in research as their developing cognitive and communicative skills may impact reliability (Borgers & Hox, 2000).

#### 3.2.0.2 IAQ modelling

For studies which modelled indoor exposure, associations were found between low household education (Rosofsky et al., 2018) and income (Shrubsole et al., 2016, Rosofsky et al., 2018) and elevated PM_2.5_ exposure. Such were the only two papers across the whole of the review which modelled indoor levels of air pollution. Shrubsole et al. (2016) used a building simulation software to predict indoor exposures from indoor and outdoor sources across representative English building archetypes. Rosofsky et al. (2018) estimated infiltration using an air exchange model, and spatial data on building properties and meteorological conditions. Despite the need to acknowledge the uncertainty inherent in model outputs, modelling techniques allow for extensive estimates of exposure and can make predictions under future scenarios, such as the effects of modifications to the building stock and future climatic variations on air quality (Shrubsole et al., 2016, Taylor et al., 2016).

For models which estimate exposure at the population level, uncertainty arises due to variation in input parameters or structural errors (Milner et al., 2011). Techniques such as sensitivity analysis allow for the relative sensitivity of the input parameters to be assessed. Further, though not present in the literature reviewed here, probabilistic models are of growing interest in IAQ modelling research as they provide the probability of a range of outcomes occurring (Dimitroulopoulou et al., 2017). Additionally, the use of empirical data, where possible, can be used to strengthen the conclusions drawn from the results.

#### 3.2.0.3 Socio-economic indicators

Capturing SES in a single metric is a complex task due to the multiple definitions the concept takes on. In the environmental tobacco literature, educational attainment was generally used as the socio-economic metric. Often, participants were asked to self-report SES information (Hughes et al., 2008, Singh et al., 2010), possibly causing a reporting bias or missing data due to the sensitive nature of the information. As a result of this, participant educational attainment was extensively used within this group of the literature.

For studies looking at exposure to indoor PM, NO_2_, radon and VOCs, household income was predominantly used as the SES metric (Son et al., 2003, Storm et al., 2013, Brown et al., 2015, Shrubsole et al., 2016, Rosofsky et al., 2018), but closely followed by household occupant density (Zota et al., 2005, Baxter et al., 2007, Brown et al., 2015) and educational attainment (Byun et al., 2010, Esplugues et al., 2010, Brown et al., 2015). Across the whole review, a small number of publications used a deprivation index (Longman and Passey, 2013, Casey et al., 2015, Shiue, 2015, Kuntz and Lampert, 2016). Composite indicators such as a deprivation indices integrate a number of different socio-economic variables into a single metric and are a response to concerns over whether a single measure can sufficiently gauge a considerably complex concept. The household deprivation index used in Kuntz & Lampert (2016) incorporated information regarding parental school education, vocational training, occupational status and household income, and is frequently used in population-level epidemiological studies in Germany (Lampert et al., 2014). SES metrics were not necessarily interchangable: Both Byun et al., 2010, Rosofsky et al., 2018 found educational inequalities were greater than income differences for PM_10_ and PM_2.5_, respectively, and exposure to indoor ETS was higher for those with a lower parental education than low household income (Bolte et al., 2008, Ren et al., 2012).

Stamatelopoulou et al. (2019) monitored particulate matter and VOCs across households, using maternal occupational status as the socio-economic metric, finding no significant difference in PM_10_, PM_2.5_ or total VOC levels in the homes of employed verses unemployed mothers. Whilst local area unemployment rates are a useful representation of neighbourhood-level deprivation, occupational status as an indicator of deprivation at the household-level may not be applicable to all members of society, for example those who are retired or students. Where information regarding employment has been readily available, male occupational status has been the preferred socio-economic indicator in epidemiological research (Galobardes et al., 2006) but parallel changes in the female labour force and shifting family dynamics mean women are now increasingly represented in the modern workforce across the developed world (The World Bank, 2018). Further evidence which suggested occupational measures may be unsuitable markers of SES was in Brown et al. (2015), who found unemployment was associated with *lower* indoor formaldehyde levels than homes with a highly skilled profession. Levels increased for all other markers of low SES (income and occupant density), suggesting that occupational measures of social position are highly circumstantial and may capture a different aspect of SES which is likely to vary between birth cohorts and family dynamics.

Correlation of SES with culture or ethnicity also emerged as a theme in the ETS literature: Gonzales et al. (2006) sampled Hispanic mothers in New Mexico, US, finding no significant difference between mothers of various SES, defined by maternal education and occupation, and childhood exposure to indoor SHS. The study found that 30% of US-born Hispanic mothers smoked, compared with 10% of Mexico-born mothers, suggesting that lower smoking rates across groups with different ethnicities may confound the relationship between exposure and deprivation as non-native subgroups of the population tend to have lower affluence than their native counterparts (Gonzales et al., 2006).

## 4 Discussion

This scoping review collates evidence on unequal exposures to indoor air pollution and justifies the incorporation of the indoor environment into the environmental equity dialogue. Understanding how indoor environmental risks are distributed across the population allows for better-targeted remedial action, improving indoor air quality and the subsequent health outcomes. It is acknowledged there are limitations in this scoping review. The search strategy focussed on a specific, though broad, range of keywords. It is accepted that some papers may have been missed as authors use titles or keywords that do not necessarily correspond with the search parameters. The review focussed only on research conducted in the developed world - it is noted though that there is a robust evidence base for exposure disparities in developing nations (WHO, 2007), which pose a significant economic burden on low-income countries (WBG, 2016).

Evidence of the exposure-deprivation relationship shows that those of lower SES were at risk of greater exposures to elevated levels of PM, NO_2_, VOCs and ETS. Indoor radon concentrations were higher in more affluent households. The review also identified a number of definitions and proxies for SES, and methods of air quality assessment. Despite the potential of housing in remedying disparate outdoor exposures through the quality of the building envelope, the evidence on indoor exposure disparities show those of low-SES were disproportionately exposed to elevated levels of indoor air pollutants. The literature suggested that policies targeting behavioural change, such as changes to public smoking legislation, can have a positive impact and extend to behaviours practised inside the home.

### 4.1 Housing interventions

Improving low-income housing standards has become a priority for many industrialised nations following the recognition of the effects of poor housing conditions on health (Ormandy, 2009), and the need to improve energy efficiency in order to reduce carbon emissions from the domestic sector (Shorrock et al., 2005). Energy-efficiency improvements in buildings are often regarded as a solution to fuel poverty in low-income housing and the associated health inequalities (Noris et al., 2013), but research has highlighted the potential detrimental effect of home retrofits on IAQ. An unintended consequence of decarbonising the built environment can be elevated exposures to indoor-sourced air pollution due to increased building airtightness: Significant increases in indoor concentrations of PM_2.5_, NO_2_ and VOCs have been observed in low-income housing following a retrofit (Broderick et al., 2017, Földváry et al., 2017). However, earlier research found levels of PM_2.5_, NO_2_, VOCs (Noris et al., 2013) and black carbon (Coombs et al., 2016), commonly caused by indoor smoking or infiltration from nearby industry and traffic (Tunno et al., 2016), were lower in retrofitted homes. An international review of indoor VOC levels (Shrubsole et al., 2019) concluded that total volatile organic compound (TVOC) concentrations in low and non-low energy homes did not vary significantly, suggesting that though low-energy buildings are generally more airtight, reducing outdoor infiltration may increase indoor-sourced pollutants, including those from building and construction materials as well as consumer products, in the absence of adequate ventilation. Research highlighting the potential trade-off between building energy efficiency and optimal IAQ has led to ventilation requirements becoming a dominant component of green building legislation (Wei et al., 2015). In the UK, the uptake of insulation and double glazing is often higher in low-income areas as a result of council-led retrofits and government schemes (Hamilton et al., 2014). Thus, the incorporation of adequate ventilation following energy-efficient building modifications is necessary to prevent poor impacts from IAQ falling disproportionately on those of low SES.

### 4.2 Monitoring IAQ

Across the review, IAQ monitoring was almost exclusively used to capture indoor exposures. For exposure to outdoor sources of air pollution, monitoring offers a simple and relatively easy method of recording outdoor levels. However, representative sample sizes can be difficult to acquire when monitoring indoor air quality, especially for domestic buildings, as this requires a high level of home-owner compliance across a number of households. It is also relatively expensive. Large variation can exist between individual dwellings as a result of the significant role of factors such as building characteristics, occupant behaviours and levels of outdoor air pollution which can modify indoor exposures significantly (Taylor et al., 2014, Fabian et al., 2016). Within a single dwelling, differences can exist between the indoor concentrations of individual rooms: Measuring indoor concentrations at fixed points within a building to estimate personal exposure may not be sufficiently representative of the actual exposure faced by the occupants, as single rooms may not be typical of indoor exposures found across the entire building (Milner et al., 2006).

To make meaningful statements about indoor exposure across SES groups, considerable sample sizes are required to ensure the sample population accurately reflects the population of interest. To isolate the experimental effect of deprivation on indoor exposure to air pollution, a control sample of both average and low-SES dwellings is required. Sample sizes can be determined via a number of approaches, including using published tables and the application of a formula. Cochran’s formula for sample size determination yields a representative sample for proportions of large populations (Cochran, 1963):

$$n_{0}=\frac{Z^{2}pq}{e^{2}}$$

Where *n*_0_ equals the sample size, Z is the z-score^3^, *p* is the estimated proportion of the total population with the attribute of interest, *q* = 1 - *p* and *e* is the desired level of precision. Where the degree of variability in the attribute of interest is unknown (p), a maximum variability of p = 0.5 is used.

Using the UK for reference, as of 2017, there were 27.2 million residential homes in the UK (ONS, 2017), 22% of which were living with relatively low-income after housing costs (DWP, 2019). Using Cochran’s formula, it would be necessary to monitor indoor exposures across a sample of 264 houses to accurately reflect exposures found in dwellings in the wider building stock with a 95% confidence interval, accounting for differences in household SES. Attaining such a sample would require an extensive monitoring campaign, with associated high costs and resources.

### 4.3 Case study – modelling IAQ

In addition to monitoring, modelling indoor air pollution levels may be achieved through various techniques, as reviewed by Milner, et al., 2004, Milner et al., 2011, including simple statistical regression (Valero et al., 2009), micro-environmental models (Dimitroulopoulou et al., 2017) and computational fluid dynamics (Panagopoulos et al., 2011). Isolating variables which contribute to indoor air pollution exposure, such as outdoor traffic density, allows for the development of targeted interventions, which often have a greater policy success on health protection. This can be difficult using monitoring methods due to the infinite variety of local circumstances, e.g. the extent of window-opening behaviour would have to be measured across all dwellings to isolate the contribution of cooking equipment on indoor levels, as ventilation helps to dissipate indoor sources of pollutants (Taylor et al., 2014).

In the UK, existing dwellings are expected to account for 70–80% of the 2050′s building stock (Palmer et al., 2011), with buildings required to undergo significant refurbishment over the next 20 years in order to meet carbon reduction targets (CCC, 2018). Modelling can identify the potential changes in IAQ caused by modification of the building stock (Shrubsole et al., 2016). Modelling is able to evaluate the relative impact of features, such as occupant behaviour (Taylor et al., 2014), on IAQ.

#### 4.3.1 Proof of concept

**Fig. 2:**
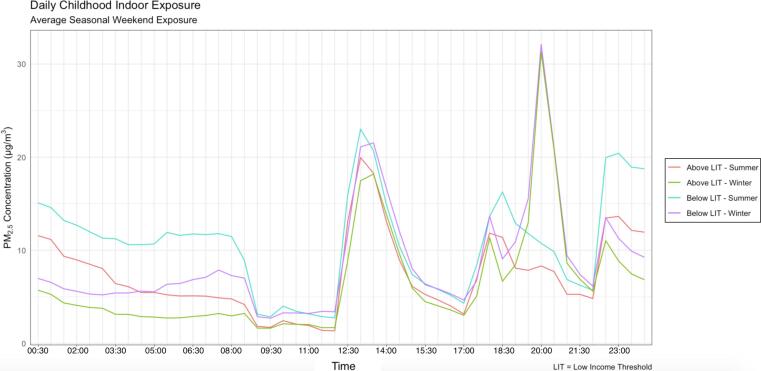
Childhood home daily PM_2.5_ exposure across households above and below the LIT, for summer and winter weekends.

To demonstrate the suitability of indoor environment modelling to the quantification of exposure disparities, a case study is outlined below. EnergyPlus (US DoE, 2014), a building physics modelling tool, was used to quantify exposures across two socio-economically different cases. Childhood home exposure to PM_2.5_, was modelled for summer and winter weekends, assuming the child was home all day, shown in Fig. 2. Children from low-income backgrounds present a *catch-22* scenario; faced with elevated exposure due to SES and increased likelihood of experiencing negative health impacts from air pollution exposure due to their immature immune and lung systems (Zhang et al., 2016). Additionally, between the ages of 7 – 12 years old, children can spend upwards of 87% of their time indoors and those younger than 3 years old may spend up to 100% of their time inside (Coombs et al., 2016), making them particularly vulnerable to indoor exposures. Occupant time-activity patterns were developed from the NatCen Time-Use Survey (Morris et al., 2016) and parameters were modelled as per Shrubsole et al. (2016), shown below in Table 7. Exposure was modelled across eight dwelling archetypes broadly representative of the English housing stock. Weighting values for each building type were inferred from the English Housing Survey (EHS, 2017), which has building composition types for households above and below the low-income threshold (LIT). In the UK, the LIT is defined as households which live on<60% of the UK’s median income (Francis-Devine et al., 2019). Windows were scheduled to open when the indoor temperature breached 25 °C.

**Table 7:** PM_2.5_ emission rates, deposition rates and production schedules for smoking and cooking.

Source	Emission rate	Deposition rate	Schedule
Cooking	1.6 mg/min^a^	0.19 h^-1^^b^	09:00 – 09:20 (kitchen)
			12:00 – 12:30 (kitchen)
			17:30 – 18:00 (kitchen)
Smoking	0.9 mg/min^a^	0.10 h^-1^^c^	12:00 – 12:05 (kitchen)
			12:30 – 12:35 (kitchen)
			17:00 – 17:05 (kitchen)
			18:00 – 18:05 (kitchen)
			10:00 – 10:05 (living room)
			11:00 – 11:05 (living room)
			15:00 – 15:05 (living room)
			16:00 – 16:05 (living room)
			19:00 – 19:05 (living room)
			20:00 – 20:05 (living room)
			21:00 – 21:05 (living room)

These production schedules were for weekends only.

a Dimitroulopoulou, Ashmore, Hill, Byrne and Kinnersley, 2006.

b Long et al., 2001.

c Klepeis and Nazaroff, 2006.

Beyond building archetypes, socio-economic information was introduced into the model via the prevalence of indoor smoking, which were weighted using empirical data from the Office of National Statistics: In 2018, 25.5% of those in routine and manual occupations smoked, compared with 15.7% of those in intermediate occupations and 10.2% of those in managerial and professional occupations (ONS, 2019). For outdoor PM_2.5_ levels, empirical monitoring data was downloaded from the London Air Quality Monitoring network for Tower Hamlets, the borough with the highest level of income inequality (Tinson et al., 2017) and Bexley, a relatively affluent borough in South East London: Across Tower Hamlets 32 middle super output areas (MSOAs)^4^, an average of 31% of households are below the LIT, compared to 8% for the equivalent figure in Bexley (Tinson et al., 2017). Results are outlined below in Fig. 2.

For both seasons, home indoor PM_2.5_ concentrations are higher in the low socio-economic case. This is due to the higher outdoor levels, indoor smoking rates and buildings with a reduced number of external façades with which to exchange outdoor air. In the early hours of the morning, concentrations are highest in the summer for both low SES homes and homes above the LIT due to the high levels of infiltration from outdoor-sourced air pollution as window opening has led to higher air exchange rates in the absence of indoor sources. Throughout the day, emissions are generated from indoor cooking and smoking activities and become higher across both socio-economic cases in the winter due to the lower air exchange rates from lower window-opening frequencies.

Whilst the model provides a useful approximation of daily exposure at home, there are limitations. A single, standard cooking profile was used – different cooking techniques can lead to appreciable differences in the amount of particulate matter emitted indoors (Abdullahi et al., 2013) - which was not accounted for in the model. A single, deterministic occupancy scenario used was assumed to be representative of childhood occupancy patterns. Whilst a deterministic approach to modelling occupant patterns and behaviour is the simplest way to integrate human behaviour into an already-complex building simulation, the approach is limited. A probabilistic approach recognises the stochasticity associated with occupant behaviour and predicts the likelihood of a range of outcomes occurring, accounting for variation in household occupation and behaviour (Aerts et al., 2014). Dimitroulopoulou et al., 2006, Dimitroulopoulou et al., 2017 used a probabilistic approach to model personal exposure of various population groups to indoor/outdoor air pollution in the home and non-residential microenvironments, using mass-balance micro-environmental models and Milner et al. (2011) highlighted the use of probabilistic time-activity information in indoor air pollution models as a priority area for future research in order to give better exposure estimates.

The tool highlights how policy interventions targeting domestic IAQ should consider the wider building and behavioural factors in a socio-economic context. Disparate indoor levels may arise due to differences in the building archetypes that are occupied by different socio-economic groups. Likewise, socially-patterned occupant behaviours can play a role: Though no link between window-opening and household income has been found (Fabi et al., 2012), those living in low SES neighbourhoods may be less likely to open windows due to low perceptions of the surrounding environment (Mavrogianni et al., 2017). While monitoring studies provide invaluable empirical evidence of exposure inequalities, modelling studies have the advantage of being able to examine a large number of different scenarios and isolate specific building and behavioural factors which may increase indoor exposures, albeit with large modelling uncertainties.

#### 4.3.2 Future work

Modelling offers a methodology through which evidence regarding adaptions to the built environment can be robustly examined before implementation in an appropriate time-frame (Hamilton et al., 2015, Shrubsole et al., 2016). Occupant behaviours such as window opening, indoor smoking prevalence and time-activity patterns can vary according to SES, but are typically excluded from building simulation software due to the difficulties generalising socially-driven behaviours. Neglecting the important interaction between building performance and household SES may result in those from a low SES background bearing a disproportionate amount of the health burden from the unanticipated effects of policies. Future work should prioritise the incorporation of qualitative information, such as household SES, into a quantitative model in order to estimate exposure disparities across income groups. Complex, multi-zonal models such as EnergyPlus, allow for exposures to be calculated for various subgroups of the population as individual factors will influence the relative proportion of time people spend in indoor micro-environments. For example, children from low-SES backgrounds may be more likely to spend time at home, watching television, due to low perceptions of the surrounding neighbourhood and a lack of after-school opportunities (Eyre et al., 2014). Calculated indoor exposures can then be applied to a parametrised stock model, such as the English Housing Survey (EHS, 2017) which gives information on household income, smoking prevalence and buildings, to calculate exposures for a representative population.

## 5 Conclusions

The work carried out has demonstrated that socio-economic inequalities in air pollution exposure extend to the indoor environment, with low-SES individuals exposed to elevated levels of indoor air pollution. Despite relatively few relevant publications, increased indoor exposure to PM, NO_2_ and VOCs fall disproportionately on populations of lower SES (Son et al., 2003, Byun et al., 2010, Brown et al., 2015), while radon was associated with higher socio-economic groups (Casey et al., 2015, Kendall et al., 2016). Exposure to environmental tobacco smoke is a problem which overwhelmingly burdens the lower social classes and the higher volumes of data in this area allow for more conclusive explanations to be drawn from the evidence (Hawkins and Berkman, 2014, Yao et al., 2016). The literature suggested that such unequal exposures may arise via poor quality housing, a lack of education regarding the harm of indoor second-hand smoke, location near congested roads and higher occupant density resulting in greater resuspension of particles. More research is needed to determine the specific mechanisms which underpin the socio-economic processes at play. Indoor environment modelling may offer a way to robustly analyse policy changes which effect the indoor environment and will play a key role in evaluating the effect of changing environmental conditions on public health.

Exposure to indoor air pollution imposes considerable health and financial burdens on developed countries (Boulanger et al., 2017). This work highlights how such burdens are likely to fall disproportionately on those of lower SES, leading to considerable health inequalities. Adoption of a holistic approach to improving indoor air quality by transforming existing cities through sustainable building design, clean household fuels and reduced dependency on cars is necessary to ensure environmental justice principles are upheld for all.

## Declaration of Competing Interest

The authors declare that they have no known competing financial interests or personal relationships that could have appeared to influence the work reported in this paper.

## Footnotes

1 Developed countries were defined as those with a score of > 0.800, indicating high human development, according to the Human Development Index (HDI) (UNDP, 2018).

2 Kendall et al. (2016) sample was taken as part of a nation-wide United Kingdom Childhood Cancer Study (UKCCS), which ran for seven years between 1991 and 1998. It assessed the effect of aetiological factors, including radon, on the health of over 11,000 children (UKCCS, 2000).

3 A *z*-score is a statistical measure characterising the spread of a dataset, used here to determine the confidence interval.

4 MSOAs are geographic areas used to improve the reporting of small area statistics. According to the 2011 census, across the UK, population per MSOA was between 5,000 and 15,000 and number of households per MSOA was 2,000 – 6,000 (ONS, 2016).

## References

[b0005] (WBG) World Bank Group (2016). The Cost of Air Pollution: Strengthening the Economic Case for Action. IHME.

[b0010] Abdullahi K.L. (2013). Emissions and indoor concentrations of particulate matter and its specific chemical components from cooking: A review. Atmos. Environ..

[b0015] Adamkiewicz G. (2011). Moving environmental justice indoors: understanding structural influences on residential exposure patterns in low-income communities. Am. J. Public Health.

[b0020] Adgate J.L. (2004). Outdoor, indoor, and personal exposure to VOCs in children. Environ. Health Perspect..

[b0025] Aerts, D., et al. (2014). “A probabilistic activity model to include realistic occupant behaviour in building simulations.” IBPSA-Canada eSim.

[b0030] Akhtar P.C. (2009). Smoking restrictions in the home and secondhand smoke exposure among primary schoolchildren before and after introduction of the Scottish smoke-free legislation. Tobacco Control.

[b0035] Akhtar P.C. (2010). Socioeconomic differences in second-hand smoke exposure among children in Scotland after introduction of the smoke-free legislation. J. Epidemiol. Community Health.

[b0040] Alwan N. (2010). Children’s exposure to second-hand smoke in the home: A household survey in the North of England. Health Soc. Care Community.

[b0045] Arku R.E. (2015). Seasonal variability in environmental tobacco smoke exposure in public housing developments. Indoor Air.

[b0050] Ashmore M., Dimitroulopoulou C. (2009). Personal exposure of children to air pollution. Atmos. Environ..

[b0055] Atkinson R. (2016). Long-term exposure to ambient ozone and mortality: a quantitative systematic review and meta-analysis of evidence from cohort studies. BMJ open.

[b0060] Batisse E. (2017). Socio-economic inequalities in exposure to industrial air pollution emissions in Quebec public schools. Can. J. Public Health.

[b0065] Baxter L.K. (2007). Predictors of concentrations of nitrogen dioxide, fine particulate matter, and particle constituents inside of lower socioeconomic status urban homes. J. Eposure Sci. Environ. Epidemiol..

[b0070] Berman B.A. (2003). Household smoking behavior and ETS exposure among children with asthma in low-income, minority households. Addict. Behav..

[b0075] Bernstein J.A. (2008). The health effects of nonindustrial indoor air pollution. J. Allergy Clin. Immunol..

[b0080] Bolte G. (2008). Socioeconomic determinants of children's environmental tobacco smoke exposure and family's home smoking policy. Europ. J. Publ. Health.

[b0085] Borgers, N. and J. Hox (2000). Reliability of responses in questionnaire research with children. fifth international conference on logic and methodology, Cologne, Germany.

[b0090] Boulanger G. (2017). Socio-economic costs of indoor air pollution: a tentative estimation for some pollutants of health interest in France. Environ. Int..

[b0095] Braubach M. (2009). Social inequalities and their influence on housing risk factors and health: a data report based on the WHO LARES database.

[b0100] Broderick Á. (2017). A pre and post evaluation of indoor air quality, ventilation, and thermal comfort in retrofitted co-operative social housing. Build. Environ..

[b0110] Brown T. (2015). Relationships between socioeconomic and lifestyle factors and indoor air quality in French dwellings. Environ. Res..

[b0115] Bruce N. (2000). Indoor air pollution in developing countries: a major environmental and public health challenge. Bull. World Health Organ..

[b0120] Byun H. (2010). Effects of socioeconomic factors and human activities on children’s PM 10 exposure in inner-city households in Korea. Int. Arch. Occup. Environ. Health.

[b0125] Casey J.A. (2015). Predictors of indoor radon concentrations in Pennsylvania, 1989–2013. Environ. Health Perspect..

[b0130] Committee on Climate Change (2018). “Reducing UK Emissions: 2018 Progress Report to Parliment ”.

[b0140] Clark L.P. (2014). National patterns in environmental injustice and inequality: outdoor NO2 air pollution in the United States.

[b0145] Clark L.P. (2017). Changes in transportation-related air pollution exposures by race-ethnicity and socioeconomic status: outdoor nitrogen dioxide in the United States in 2000 and 2010. Environ. Health Perspect..

[b0150] Cochran W.G. (1963). Sampling Techniques.

[b0155] COMEAP (2018). Associations of long-term average concentrations of nitrogen dioxide with mortality. A report by the Committee on the Medical Effects of Air Pollutants.

[b0160] COMEAP (2018). The Effects of Long-Term Exposure to Ambient Air Pollution on Cardiovascular Morbidity: Mechanistic Evidence.“ Committee on the Medical Effects of Air Pollutants.

[b0710] Common Communication Format Standardization (1993). “Workplace atmospheres - size fraction definitions for measurement of airborne particles”. British Standards Institute.

[b0170] Coombs K.C. (2016). Indoor air quality in green-renovated vs. non-green low-income homes of children living in a temperate region of US (Ohio). Sci. Total Environ..

[b0175] DEFRA (2019). “Open fires and wood burning stoves - a pratical guide.” Department for Environment, Food and Rural Affairs.

[b0180] Deguen S., Zmirou-Navier D. (2010). Social inequalities resulting from health risks related to ambient air quality—a European review. Eur. J. Pub. Health.

[b0185] Delgado-Saborit J.M. (2012). Use of real-time sensors to characterise human exposures to combustion related pollutants. J. Environ. Monit..

[b0190] Dimitroulopoulou C. (2006). INDAIR: a probabilistic model of indoor air pollution in UK homes. Atmos. Environ..

[b0195] Dimitroulopoulou C. (2015). EPHECT II: Exposure assessment to household consumer products. Sci. Total Environ..

[b0200] Dimitroulopoulou C. (2017). Use of population exposure frequency distributions to simulate effects of policy interventions on NO2 exposure. Atmos. Environ..

[b0210] D'souza, J. C., et al. (2009). “Ethnicity, housing and personal factors as determinants of VOC exposures.” Atmosph. Environ. 43(18): 2884-2892.

[b0215] Dumanoglu Y. (2014). Spatial and seasonal variation and source apportionment of volatile organic compounds (VOCs) in a heavily industrialized region. Atmos. Environ..

[b0220] (DWP) Department for Working Pensions (2019). “Households Below Average Income: An analysis of the UK income distribution: 1994/95-2017/18.” Office for National Statistics.

[b0225] European Environment Agency (2019). “Unequal exposure and unequal impacts: social vulnerability to air pollution, noise and extreme temperatures in Europe.” EEA Report No 22/2018.

[b0230] EHS (2017). “English Housing Survey.” Department for Communities and Local Government Headline Report, 2015-2016.

[b0235] (EPA) Environmental Protection Agency (2016). “Volatile Organic Compounds’ Impact on Indoor Air Quality”.

[b0240] Esplugues A. (2010). Indoor and outdoor concentrations and determinants of NO2 in a cohort of 1-year-old children in Valencia, Spain. Indoor Air.

[b0245] Eyre E.L.J. (2014). Low socio-economic environmental determinants of children's physical activity in Coventry, UK: A Qualitative study in parents. Prevent. Med. Rep..

[b0250] Fabi V. (2012). Occupants' window opening behaviour: A literature review of factors influencing occupant behaviour and models. Build. Environ..

[b0255] Fabian M. (2016). Modeling environmental tobacco smoke (ETS) infiltration in low-income multifamily housing before and after building energy retrofits. Int. J. Environ. Res. Publ. Health.

[b0260] Fairburn J. (2019). Social inequalities in exposure to ambient air pollution: a systematic review in the WHO European region. Int. J. Environ. Res. Public Health.

[b0265] Ferrero A. (2017). Infants' indoor and outdoor residential exposure to benzene and respiratory health in a Spanish cohort. Environ. Pollut..

[b0275] Földváry V. (2017). Effect of energy renovation on indoor air quality in multifamily residential buildings in Slovakia. Build. Environ..

[b0280] Foulds C. (2013). Investigating the performance of everyday domestic practices using building monitoring. Build. Res. Informat..

[b0285] Francis-Devine, B. B., Lorna; McGuinness, Feargal (2019). “Poverty in the UK: Statistics ” House of Commons Library Briefing Paper: Number 7096.

[b0290] Fuller G. (2019). The Invisible Killer: The Rising Global Threat of Air Pollution-and how We Can Fight.

[b0295] Galobardes B. (2006). Indicators of socioeconomic position (part 2). J. Epidemiol. Community Health.

[b0300] Géhin E. (2008). Size distribution and emission rate measurement of fine and ultrafine particle from indoor human activities. Atmos. Environ..

[b0305] Gligorovski S. (2016). Nitrous acid (HONO): An emerging indoor pollutant. J. Photochem. Photobiol., A.

[b0310] Goldemberg J. (2018). Household air pollution, health, and climate change: cleaning the air. Environ. Res. Lett..

[b0315] Gonzales M. (2006). Prevalence and predictors of home and automobile smoking bans and child environmental tobacco smoke exposure: a cross-sectional study of US-and Mexico-born Hispanic women with young children. BMC Public Health.

[b0320] Hajat A. (2015). Socioeconomic disparities and air pollution exposure: a global review. Curr. Environ. Health Rep..

[b0325] Hamilton I.G. (2014). Uptake of energy efficiency interventions in English dwellings. Build. Res. Inform..

[b0330] Hamilton I. (2015). Health effects of home energy efficiency interventions in England: a modelling study. BMJ open.

[b0335] Hänninen O. (2004). Infiltration of ambient PM2. 5 and levels of indoor generated non-ETS PM2. 5 in residences of four European cities. Atmos. Environ..

[b0345] Hawkins S., Berkman L. (2014). Identifying infants at high-risk for second-hand smoke exposure. Child Care Health Dev..

[b0350] Hughes S.C. (2008). Children’s exposure to second hand smoke at home in Seoul, Korea. Asian Pac. J. Cancer Prev..

[b0355] ISO (2009). “23210: 2009 Stationary source emissions-Determination of PM10.” PM2, 5 mass concentration in flue gas-Measurement at low concentrations by use of impactors (ISO 23210: 2009).

[b0360] Jacobs D.E. (2011). Environmental health disparities in housing. Am. J. Public Health.

[b0365] Jarvis MJ, M. J., Gillmore A, et al. (2009). “Smokefree homes in England: prevalence, trends and validation by cotinine in children.” Tobacco Control: 18:491–495. 10.1136/tc.2009.031328 19748885

[b0370] Jurado D. (2004). Environmental tobacco smoke exposure in children: parental perception of smokiness at home and other factors associated with urinary cotinine in preschool children. J. Eposure Sci. Environ. Epidemiol..

[b0375] Kendall G.M. (2016). Variation with socioeconomic status of indoor radon levels in Great Britain: the less affluent have less radon. J. Environ. Radioact..

[b0380] Klepeis N.E., Nazaroff W.W. (2006). Modeling residential exposure to secondhand tobacco smoke. Atmos. Environ..

[b0385] Kuntz B., Lampert T. (2016). Social disparities in parental smoking and young children’s exposure to secondhand smoke at home: a time-trend analysis of repeated cross-sectional data from the German KiGGS study between 2003–2006 and 2009–2012. BMC Publ. Health.

[b0390] Lampert T. (2014). Measurement of socioeconomic status in the KiGGS study: first follow-up (KiGGS Wave 1). Bundesgesundheitsblatt, Gesundheitsforschung, Gesundheitsschutz.

[b0395] Liao Y.-M. (2013). Factors associated with parental smoking in the presence of school-aged children: a cross-sectional study. BMC Publ. Health.

[b0400] Long C.M. (2001). Using time-and size-resolved particulate data to quantify indoor penetration and deposition behavior. Environ. Sci. Technol..

[b0405] Longman J.M., Passey M.E. (2013). Children, smoking households and exposure to second-hand smoke in the home in rural Australia: analysis of a national cross-sectional survey. BMJ open.

[b0410] Loomis D. (2013). The carcinogenicity of outdoor air pollution. Lancet Oncol..

[b0415] Ma Q. (2017). SO2 initiates the efficient conversion of NO2 to HONO on MgO surface. Environ. Sci. Technol..

[b0420] Mannino D.M. (2001). Predictors of cotinine levels in US children: data from the third national health and nutrition examination survey. Chest.

[b0425] Mantziou V. (2009). Predictors of childhood exposure to parental secondhand smoke in the house and family car. Int. J. Environ. Res. Public Health.

[b0435] Mavrogianni A. (2017). Inhabitant actions and summer overheating risk in London dwellings. Building Research & Information.

[b0440] McGrath J. (2017). PM exposure variations due to different time activity profile simulations within a single dwelling. Build. Environ..

[b0450] Milner J.T. (2006). Spatial variation of CO concentrations within an office building and outdoor influences. Atmos. Environ..

[b0455] Milner J. (2011). Modelling inhalation exposure to combustion-related air pollutants in residential buildings: application to health impact assessment. Environ. Int..

[b0460] Milner, J., et al. (2004). “Indoor concentrations in buildings from sources outdoors.” ADMLC Annual Report 2005.

[b0465] Milojevic, A., et al. (2017). “Socioeconomic and urban-rural differentials in exposure to air pollution and mortality burden in England.” 16(1): 104. 10.1186/s12940-017-0314-5 PMC6389046 28985761

[b0470] Morris, S., et al. (2016). “The UK Time Diary Study 2014–2015”.

[b0475] Moulton Paula Valencia, Yang Wei (2012). Air pollution, oxidative stress, and alzheimer's disease. J. Environ. Publ. Health.

[b0480] Murage, P., et al. (2020). “What individual and neighbourhood-level factors increase the risk of heat-related mortality? A case-crossover study of over 185,000 deaths in London using high-resolution climate datasets.” Environ. Int. 134: 105292. 10.1016/j.envint.2019.105292 PMC7103759 31726356

[b0485] Nguyen M. (2018). Passive smoking at home by socioeconomic factors in a japanese population: NIPPON DATA2010. J. Epidemiol..

[b0490] NHS (2019). “Statistics on Smoking, England – National Statistics [PAS]”.

[b0495] (NICE) National Institute for Health and Care Excellence (2013). “Smoking: harm reduction ” NICE Guidance: Lifestyle and Wellbeing: Smoking and tobacco Public health guideline [PH45].

[b0500] Noris F. (2013). Indoor environmental quality benefits of apartment energy retrofits. Build. Environ..

[b0505] Noutsios G.T., Floros J. (2014). Childhood asthma: causes, risks, and protective factors; a role of innate immunity. Swiss Medical Weekly.

[b0510] O'Leary C. (2019). Setting the standard: the acceptability of kitchen ventilation for the English housing stock. Build. Environ..

[b0515] (ONS) Office for National Statistics (2016). “Census geography ”.

[b0520] (ONS) Office for National Statistics (2017). “Families and Households: 2017”.

[b0525] (ONS) Office for National Statistics (2019). “Adult smoking habits in the UK: 2018.” Statistical Bull.

[bib841] Ormandy David, World Health Organisation (2009). Housing and Health in Europe: The WHO Lares Project.

[b0530] Orton S. (2014). Predictors of children's secondhand smoke exposure at home: a systematic review and narrative synthesis of the evidence. PLoS ONE.

[b0535] Padilla, C. M., et al. (2014). “Air quality and social deprivation in four French metropolitan areas—A localized spatio-temporal environmental inequality analysis.” 134: 315-324. 10.1016/j.envres.2014.07.017 PMC4294705 25199972

[b0540] Padula A.M. (2013). Ambient air pollution and traffic exposures and congenital heart defects in the S an J oaquin Valley of C alifornia. Paediatr. Perinat. Epidemiol..

[b0545] Palmer, J. and I. Cooper (2013). “United Kingdom housing energy fact file 2013.” Department of Energy & Climate Change, Prepared under contract to DECC by Cambridge Architectural Research, Eclipse Research Consultants and Cambridge Energy. The views expressed are not necessarily DECC’sp 172.

[b0550] Palmer, J., et al. (2011). “Great Britain’s housing energy fact file 2011.” Report, Department of Energy and Climate Change, UK.

[b0555] Panagopoulos I.K. (2011). A CFD simulation study of VOC and formaldehyde indoor air pollution dispersion in an apartment as part of an indoor pollution management plan. Aerosol Air Quality Res..

[b0560] Park M. (2013). Effects of air pollution on asthma hospitalization rates in different age groups in metropolitan cities of Korea. Air Qual. Atmos. Health.

[b0565] Patino E.D.L., Siegel J.A. (2018). Indoor environmental quality in social housing: a literature review. Build. Environ..

[b0570] (PHE) Public Health England (2018). “Health matters: stop smoking - what works?” Guidance.

[b0575] Pinault L. (2016). Socioeconomic differences in nitrogen dioxide ambient air pollution exposure among children in the three largest Canadian cities.

[b0580] Pisinger C. (2012). Social disparities in children’s exposure to second hand smoke at home: a repeated cross-sectional survey. Environ. Health.

[b0585] Poljansek, K., et al. (2017). Science for disaster risk management 2017: knowing better and losing less, ETH Zurich.

[b0590] Pope C.A. (2011). Lung cancer and cardiovascular disease mortality associated with ambient air pollution and cigarette smoke: shape of the exposure–response relationships. Environ. Health Perspect..

[b0600] Pye S. (2001). Further analysis of NO2 and PM10 air pollution and social deprivation.

[b0605] Raisamo S.U. (2014). Persistence of socioeconomic differences in adolescents’ environmental tobacco smoke exposure in Finland: 1991–2009. Scandinavian J. Publ. Health.

[b0610] Ren Y. (2012). Are urban low-income children from unplanned pregnancy exposed to higher levels of environmental tobacco smoke?. J. Pediatric Health Care.

[b0615] Ribas-Fitó N. (2006). Child health and the environment: the INMA Spanish Study. Paediatr. Perinat. Epidemiol..

[b0620] Rise J., Lund K.E. (2005). Predicting children's level of exposure to environmental tobacco smoke based on two national surveys in Norway in 1995 and 2001. Addict. Behav..

[b0630] Rosofsky A. (2018). The impact of air exchange rate on ambient air pollution exposure and inequalities across all residential parcels in Massachusetts. J. Exposure Sci. Environ.Epidemiol..

[b0635] Samoli E. (2019). Spatial variability in air pollution exposure in relation to socioeconomic indicators in nine European metropolitan areas: A study on environmental inequality. Environ. Pollut..

[b0640] Scherer G. (2004). Determinants of children's exposure to environmental tobacco smoke (ETS): a study in Southern Germany. J. Eposure Sci. Environ. Epidemiol..

[b0645] Schmidt C.W. (2007). A change in the air: smoking bans gain momentum worldwide. Environ. Health Perspectives.

[b0650] Shiue I. (2015). Correlations of indoor second-hand smoking, household smoking rules, regional deprivation and children mental health: Scottish Health Survey, 2013. Environ. Sci. Pollut. Res..

[b0595] (UNDP) United Nations Development Programme (2018). “Human Development Indices and Indicators ” 2018 Statistical Update.

[b0655] Shorrock, L., et al. (2005). “Reducing carbon emissions from the UK housing stock.” BRE Report BR480.

[b0660] Shrubsole C. (2014). 100 Unintended consequences of policies to improve the energy efficiency of the UK housing stock. Indoor Built Environ..

[b0665] Shrubsole C. (2016). Impacts of energy efficiency retrofitting measures on indoor PM2. 5 concentrations across different income groups in England: a modelling study. Adv. Build. Energy Res..

[b0670] Shrubsole, C., et al. (2019). “IAQ guidelines for selected volatile organic compounds (VOCs) in the UK.” Building and Environment: 106382.

[b0675] Simons E., To T., Moineddin R., Stieb D., Dell S.D. (2014). Maternal second-hand smoke exposure in pregnancy is associated with childhood asthma development. J. Allergy Clin. Immunol. Pract..

[b0680] Singer B.C. (2017). Pollutant concentrations and emission rates from natural gas cooking burners without and with range hood exhaust in nine California homes. Build. Environ..

[b0685] Singh G.K. (2010). Disparities in children's exposure to environmental tobacco smoke in the United States, 2007. Pediatrics.

[b0690] Smith K.R., Mehta S. (2003). The burden of disease from indoor air pollution in developing countries: comparison of estimates. Int. J. Hyg. Environ. Health.

[b0695] Soliman S. (2004). Decrease in the prevalence of environmental tobacco smoke exposure in the home during the 1990s in families with children. Am. J. Public Health.

[b0700] Son B. (2003). Volatile organic compounds concentrations in residential indoor and outdoor and its personal exposure in Korea. Environ. Int..

[b0705] Stamatelopoulou A. (2019). Effects of PM, TVOCs and comfort parameters on indoor air quality of residences with young children. Build. Environ..

[b0715] Storm J.E. (2013). Socioeconomic disparities in indoor air, breath, and blood perchloroethylene level among adult and child residents of buildings with or without a dry cleaner. Environ. Res..

[b0720] Symonds P. (2019). Home energy efficiency and radon: an observational study. Indoor Air.

[b0725] Taylor J. (2014). The modifying effect of the building envelope on population exposure to PM 2.5 from outdoor sources. Indoor Air.

[b0730] Taylor J. (2016). Mapping indoor overheating and air pollution risk modification across Great Britain: A modelling study. Build. Environ..

[b0735] The World Bank (2018). “Female Labor.

[b0740] Thomson G. (2005). Smoky homes: a review of the exposure and effects of secondhand smoke in New Zealand homes. The New Zealand Medical Journal (Online).

[b0745] Tinson, A., et al. (2017). “London’s Poverty Profile.” Trust for London.

[b0750] Tonne C. (2008). Air pollution and mortality benefits of the London Congestion Charge: spatial and socioeconomic inequalities. Occup. Environ. Med..

[b0755] Tricco A.C. (2018). PRISMA extension for scoping reviews (PRISMA-ScR): checklist and explanation. Ann. Intern. Med..

[b0760] Tunno B.J. (2016). Indoor source apportionment in urban communities near industrial sites. Atmos. Environ..

[b0765] Turk B.H. (1990). Characterizing the occurrence, sources, and variability of radon in Pacific Northwest homes. J. Air Waste Manag. Assoc..

[b0770] UKCCS (2000). “The United Kingdom childhood cancer study: objectives, materials and methods.” British Journal of Cancer 82(5): 1073. 10.1054/bjoc.1999.1045 PMC2374433 10737392

[b0775] Ulbricht S. (2014). Predictors of indoor smoking at young children’s homes—a cross-sectional study. Eur. J. Pediatr..

[b0780] U.S. Department of Energy (2014). “EnergyPlus Testing with Building Thermal Envelope and Fabric Load Tests from ANSI/ASHRAE Standard 140-2011.” EnergyPlus Version 8.2.0.

[b0785] Valero N. (2009). Concentrations and determinants of outdoor, indoor and personal nitrogen dioxide in pregnant women from two Spanish birth cohorts. Environ. Int..

[b0790] Wang S.-W. (2009). Characterizing relationships between personal exposures to VOCs and socioeconomic, demographic, behavioral variables. Atmos. Environ..

[b0795] Wei W. (2015). Indoor air quality requirements in green building certifications. Build. Environ..

[b0800] Organisation, W. H. (2007). “Indoor Air Pollution: National Burden of Disease Estimates.” WHO, Geneva, Switzerland.

[b0805] Organization, W. H. (2019). “Environmental health inequalities in Europe.” Public Health.

[b0815] Yao T. (2016). Sociodemographic differences among US children and adults exposed to secondhand smoke at home: National Health Interview Surveys 2000 and 2010. Public Health Rep..

[b0820] Yi O. (2011). Association between environmental tobacco smoke exposure of children and parental socioeconomic status: a cross-sectional study in Korea. Nicotine Tob. Res..

[b0825] Zhang S. (2016). Interventions to reduce individual exposure of elderly individuals and children to haze: a review. J. Thorac. Dis..

[b0830] Zheng X.-Y. (2015). Association between air pollutants and asthma emergency room visits and hospital admissions in time series studies: a systematic review and meta-analysis. PLoS ONE.

[b0835] Zipprich J.L. (2002). An analysis of factors that influence personal exposure to nitrogen oxides in residents of Richmond, Virginia. J. Eposure Sci. Environ. Epidemiol..

[b0840] Zota A., Adamkiewicz G., Levy J.I., Spengler J.D. (2005). Ventilation in public housing: Implications for indoor nitrogen dioxide concentrations. Indoor Air.

